# Mitigating drought-induced oxidative stress in wheat (*Triticum aestivum* L.) through foliar application of sulfhydryl thiourea

**DOI:** 10.1038/s41598-024-66506-y

**Published:** 2024-07-10

**Authors:** Nazia Ishfaq, Ejaz Ahmad Waraich, Muhammad Ahmad, Saddam Hussain, Usman Zulfiqar, Kaleem Ul Din, Arslan Haider, Jean Wan Hong Yong, Syed Muhammad Hassan Askri, Hayssam M. Ali

**Affiliations:** 1 Department of Agronomy, University of Agriculture, Faisalabad, 38040 Pakistan; 2 Department of Agronomy, Faculty of Agriculture and Environment, The Islamia University of Bahawalpur, Bahawalpur, 63100 Pakistan; 3 Department of Botany, University of Agriculture, Faisalabad, 38040 Pakistan; 4 Department of Biosystems and Technology, Swedish University of Agricultural Sciences, Alnarp, 23456 Sweden; 5 Zhejiang Key Laboratory of Crop Germplasm Resource, Department of Agronomy, College of Agriculture and Biotechnology, Zhejiang University, Hangzhou, 310058 China; 6 Department of Botany and Microbiology, College of Science, King Saud University, 11451 Riyadh, Saudi Arabia

**Keywords:** Drought stress, Antioxidants activities, Malondialdehyde production, Osmolytes, Physiology, Plant sciences

## Abstract

Drought stress is a major abiotic stress affecting the performance of wheat (*Triticum aestivum* L*.*). The current study evaluated the effects of drought on wheat phenology, physiology, and biochemistry; and assessed the effectiveness of foliar-applied sulfhydryl thiourea to mitigate drought-induced oxidative stress. The treatments were: wheat varieties; V_1_ = Punjab-2011, V_2_ = Galaxy-2013, V_3_ = Ujala-2016, and V_4_ = Anaaj-2017, drought stress; D_1_ = control (80% field capacity [FC]) and D_2_ = drought stress (40% FC), at  the reproductive stage, and sulfhydryl thiourea (S) applications; S_0_ = control-no thiourea and S_1_ = foliar thiourea application @ 500 mg L^−1^. Results of this study indicated that growth parameters, including height, dry weight, leaf area index (LAI), leaf area duration (LAD), crop growth rate (CGR), net assimilation rate (NAR) were decreased under drought stress-40% FC, as compared to control-80% FC. Drought stress reduced the photosynthetic efficiency, water potential, transpiration rates, stomatal conductances, and relative water contents by 18, 17, 26, 29, and 55% in wheat varieties as compared to control. In addition, foliar chlorophyll a, and b contents were also lowered under drought stress in all wheat varieties due to an increase in malondialdehyde and electrolyte leakage. Interestingly, thiourea applications restored wheat growth and yield attributes by improving the production and activities of proline, antioxidants, and osmolytes under normal and drought stress as compared to control. Thiourea applications improved the osmolyte defense in wheat varieties as peroxidase, superoxide dismutase, catalase, proline, glycine betaine, and total phenolic were increased by 13, 20, 12, 17, 23, and 52%; while reducing the electrolyte leakage and malondialdehyde content by 49 and 32% as compared to control. Among the wheat varieties, Anaaj-2017 showed better resilience towards drought stress and also gave better response towards thiourea application based on morpho-physiological, biochemical, and yield attributes as compared to Punjab-2011, Galaxy-2013, and Ujala-2016. Eta-square values showed that thiourea applications, drought stress, and wheat varieties were key contributors to most of the parameters measured. In conclusion, the sulfhydryl thiourea applications improved the morpho-physiology, biochemical, and yield attributes of wheat varieties, thereby mitigating the adverse effects of drought.  Moving forward, detailed studies pertaining to the molecular and genetic mechanisms under sulfhydryl thiourea-induced drought stress tolerance are warranted.

## Introduction

Wheat is an important cereal which is a healthy nutritional source predominantly in a resource-depriving world. In the physical year 2021–2022, the total wheat production was 778.6 million tons providing 40% of protein and carbohydrate-rich food requirement around the world ^1,2^. In Asia, wheat is the most widely consumed cereal crop, and it is a significant crop in Pakistan as well ^3–5^. When compared to other cereal crops, wheat is the best source of protein, accounting for 19% of global dietary energy and 21% of overall protein consumption ^6^. Factors like drought, temperature rise, and climate change significantly reduce wheat yield, posing a serious concern given the growing global population's increasing demand^7,8^. Among these, drought poses a serious risk to wheat production, directly impacting crop productivity^9–11^. Water shortage in the soil lead to decreased nutrient uptake by the roots, which is directly linked to the reduced stomatal conductance and transpiration rates^12,13^.

Worldwide water scarcity is expected to prevail for a longer duration^14^ and causing drought stress that adversely affects the production of crops of the winter season like wheat^15^. Drought stress becomes the major issue of wheat production that can lead to food insecurity in the arid and semi-arid of the world. In developing countries such as Pakistan, agricultural production is dependent on the canal irrigation system for water supplies during designated periods in tandem with relevant crop cultivation schedules^16^. Pakistan is a country that is often affected by drought episodes^17^. It was discovered that about 33% of all arable land is susceptible to drought, which is a major problem that lowers the productivity and output of cereals^18^. Drought stress affects numerous physiological processes such as membrane integrity, photosynthesis, growth, pollen viability, abscisic acid, and proline concentration^19^. Wheat is particularly sensitive to temperature stress during the reproductive stage and also to drought stress during the vegetative stage, both of which can lower the crop performance^20^. Drought stress creates osmotic imbalance and disturbs the membrane stability may damage the increase in the leakage of metabolites and cause cell death^21^. Based on the stage of crop, duration, and severity of drought, the yield of wheat was reduced by up to 90% due to the stress-induced damage to photosynthetic machinery and plant metabolism^22^. Plants experiencing drought may exhibit various altered states, such as decreased rates of photosynthesis, reduced fresh and dry biomass, growth, and decreased uptake of vital nutrients from the soil^23–25^. The primary causes of plant damage resulting from drought-induced highly reactive oxygen species (ROS) generation are reductions in chlorophyll content and oxidation of membrane lipids and proteins, which alter the cellular redox status^12^. Under drought stress, excessive ROS production leads to lipid peroxidation of cell membranes, resulting in reduced plant phenology and physiology^26^. Therefore, increasing wheat production requires enhancing its capacity to withstand stress and understanding the mechanisms by which the plants adapt to drought conditions^27^. The effects of drought stress depends upon the length of the crop period, the severity of the drought, and environmental conditions ^28^. Interestingly, plants have strong antioxidative defense mechanisms to overcome and repair damage through oxidative stress and glyoxalase detoxification mechanisms^29^. Antioxidant enzymes, such as glutathione reductase (GSH), catalase (CAT), superoxide dismutase (SOD), and ascorbate peroxidase (AsA), are essential to the plant defense mechanism against drought stress because they scavenge excessive ROS^30^. Drought tolerance is the ability of a plant to continue growing and developing even in the context of water scarcity^25,31^. Thus, enhancing the ability of wheat to tolerate drought stress is crucial for sustaining physiological resilience, continual crop production and ensuring food security ^32^.

In addition to the natural defense mechanisms evolved by plants, they can be protected from drought stress by applying exogenous substances, mineral nutrients, biostimulants and microorganisms by upregulating the different physicochemical mechanisms at the metabolic and whole-plant levels ^33–37^. The essential nutrients/plant growth regulators, including sulfhydryl thiourea, help to ameliorate the negative effects of abiotic stress; restoring the photosynthetic processes, especially during unfavorable conditions ^38,39^. Thiourea functions as a non-physiological thiol and is a highly effective radicle scavenger ^38–41^. Several intricate mechanisms are necessary for abiotic stress tolerance, and thiourea may control several of them ^42^. Thiourea significantly increases the nutritional and quality status of wheat grains by increasing the percentage of oil, soluble sugars, free amino acids, and protein content^43^. Applying thiourea as a foliar spray improved plant phenology, photosynthetic pigments, and yield characteristics, significantly mitigating drought stress's negative effects^44^. Thiourea, a non-physiological ROS scavenger, is considered as an effective drought alleviator because of its well-proven effectiveness in the field, favorable benefit-to-cost ratio, safety for the environment, and acceptance by consumers^45^. Through its modulation of transporters, defense systems, nutritional homeostasis, and metabolic activities, thiourea promotes the reductive cellular environment during stress^46^. Applications of thiourea boost the activities of antioxidants and plant osmolytes that may help the plant upregulate its defense system against drought-induced oxidative stress^47^. We must evaluate the newest methods for producing and managing wheat to handle the difficulties of feeding a growing population in demanding areas, particularly in light of anticipated climate change. The goal of the current study is to assess the impact of thiourea on the physiological and biochemical characteristics of wheat during drought stress, which suggests that our understanding of the underlying genetic controls and signaling systems is still lacking. Nonetheless, there is an urgent need to investigate how STU applications affect plant signaling pathways and how they function at the cellular level, which emphasizes the need for additional investigation to clarify the genetic underpinnings of thiourea-induced drought stress tolerance in plants.

The studies on the use of sulfur-rich thiourea have been documented. In different crops, such as camelina^41^, canola^47^, and maize^48^, sulfur-rich thiourea has been shown to improve plant growth, while the role of thiourea in alleviating drought stress in wheat hasn't been studied yet. However, there is an urgent need to evaluate the effect of sulfhydryl thiourea application to mitigate drought-induced oxidative stress in wheat. The study hypothesized that the exogenous applications of sulfur-rich thiourea may improve the resilience of wheat grown under drought stress. However, the objective of the present study was to evaluate the role of sulfur-rich thiourea in the amelioration of negative impacts of drought stress by improving the plant's physiological attributes, osmolyte production, and antioxidant activities in wheat varieties.

## Materials and methods

### Experimental material and experimental conditions

The study was conducted in the wire-house in the Department of Agronomy, University of Agriculture, Faisalabad. The wire-house was covered with a strong sheet to protect the experimental pots from rainfall. Seeds of four wheat varieties (Punjab 2011, Galaxy 2013, Ujala 2016, and Anaj 2017) were collected from Ayub Agricultural Research Institute, Faisalabad. The treatments were: wheat varieties; V_1_ = Punjab-2011, V_2_ = Galaxy-2013, V_3_ = Ujala-2016, and V_4_ = Anaaj-2017, drought stress; D_1_ = control (80% field capacity (FC)) and D_2_ = drought stress (40% FC) at the vegetative stage, and sulfhydryl thiourea applications; S_0_ = control-no thiourea and S_1_ = foliar thiourea application at 500 mg L^−1^ at 30 DAS under a Completely Randomized Design and factorial arrangements with three replications. The gravimetric method was used to measure the FC of sand^49^. The sowing was done in a total of 48 weighed pots (28 × 22 cm) and each pot was filled with 4 kg of sand. Twelve seeds of wheat varieties were sown in each pot at 80% FC and maintained at only 8 plants per pot by thinning at 15 days after sowing (DAS) [4 leaf stage-BBCH Scales, 42,43. The moisture status was checked and maintained daily by weighting the pots. At the principal growth stage 4: Booting [40 DAS-BBCH Scale Code-40, 42,43, the drought stress was applied at 40% FC for 14 days in half 24 pots and the other 24 pots were under normal conditions-80% FC The thiourea at 500 mg L^−1^ (12 mL pot ^−1^) was mixed with distilled water and the solution was foliarly sprayed twice at booting stage of wheat at 40 DAS and 45 DAS. Hoagland solution was applied at sowing and then after every fortnight to provide proper nutrition to the wheat crop.

### Periodic data collection

The growth and physiological data were taken periodically at 50 DAS, 60 DAS, 70 DAS, 80 DAS, and 90 DAS. The biochemical attributes were taken at 80 DAS and 90 DAS and averaged.

## Observations

### Growth parameters

For the various growth parameters, three plants from each pot were randomly selected and plant height was measured by using a meter rod at every periodic interval. The samples were oven (Memmert-110, Schawabach, Germany) dried at 65°C and electrical balance (Kern 440-49A, Balingen, Germany) was used to record the dry weight. LAI-2200C Plant Canopy Analyzer was used to measure the leaf area index (LAI) of wheat plants. LAD, CGR, and NAR were measured by using standard procedures^50–53^.

### Physiological parameters

The relative water contents (RWC) were calculated according to the procedure pursued by Ahmad et al.^33^, whereas the water potential was measured by using a pressure bomb (ARIMAD-2, ELE- International, Japan).

Gas exchange parameters of wheat leaves were taken by using a gas exchange system, with an infrared gas analyzer (CI-340 portable, Hoddesdon, England) from 10 to 11 am under standard conditions as described by Ahmad et al.^33^; the gas exchange calculations were in accordance to Farquhar et al.^54^.

### Chlorophyll contents

The chlorophyll contents of wheat plants were measured by using the procedure of Arnon^55^ and was followed with minor modifications^56^. The top 3^rd^ leaf of wheat plants was taken from every biological replicate and placed in vials of 15 mL and acetone (10 mL) was added. A spectrophotometer (IRMECO U2020, Geesthacht/Germany) was used to measure absorbance at 645 and 665 nm.

### Stress indicators

Membrane stability was tested by measuring the electrolyte leakage by following the procedure given by Dionisio-Sese and Tobita^57^. Cakmak and Horst^58^ gave the protocol to measure the malondialdehyde (MDA) contents. The fresh wheat samples of weight 0.5 g were ground in a solution of trichloroacetic acid solution (10 mL) and centrifuged for 15 min. at 12,000 × g. In 1 mL of supernatant, 4 mL of trichloroacetic acid (0.5%) was added and heated for 30 min. in a water bath at 95 °C. A spectrophotometer was used to measure optical density (OD) at 532 and 600 nm against a blank consisting of trichloroacetic acid (5%).

### Antioxidants activities

The activity of superoxide dismutase was estimated by the process of Dhindsa et al.^59^. Wheat leaves (200 mg) were homogenized in an extraction buffer of 0.1 M phosphate (2 mL), 0.5 mM of EDTA + 7.5 pH, centrifuged at 10,000 × g and stored at 4 °C. Spectrophotometer was used to measure the absorbance at 560 nm. Putter^60^ defined the process to measure the activity of peroxidase. The reaction mixture containing 10 mM of guaiacol, 50 mM of phosphate buffer at pH 7.0, and 5 mM of H_2_O_2_ was preheated in a water bath at 20 °C. After that, the supernatant and enzyme were thoroughly mixed in added in a centrifuged tube. A spectrophotometer was used to measure the absorbance at 470 nm.

Catalase activity was measured by following the procedure of Liu et al.^61^. The enzyme extract was mixed with the freshly primed 5.9 mM hydrogen peroxide (35% pure, 100 µL) at the start of the reaction. The rate of disappearance of hydrogen peroxide was recorded by using a microplate reader (ELX800, Bio-Tek Instruments, Inc., Winooski, VT, USA) at 240 nm that witnessed the reduction in the absorbance at the wavelength of 240 nm for 3 min. to measure catalase.

### Osmolyte/Metabolite production

The wheat samples were homogenized in 5-sulfosalicylic acid (3%) to measure proline (Pr) content. Then, glacial acetic acid and acid ninhydrin were mixed with sample extracts. The reaction mixture was mixed with toluene and vigorously shaken for 25 s, and proline content was recorded defined by Bates et al.^62^. Glycine betaine (GB) content was measured by extracting fresh wheat samples in deionized-water and sulfuric acid (2 mM) by following the procedure defined by Grieve and Grattan^63^.

The Folin-Ciocalteu technique was used to quantify the total soluble phenolics in acetone extract^64^. The wheat samples that were specifically weighed were mashed in acetone (80%), and then centrifuged at 1000 × g for 15 min. The 0.1 mL supernatant was then combined with 2 mL water and 1 mL Folin–Ciocalteau phenol reagent. To 5 mL of Na_2_CO_3_, 10 mL of distilled water was added (20%). Using a spectrophotometer, the OD at 750 nm was measured.

### Yield parameters

The yield and yield parameters were measured at 145 DAS. To measure number of productive tillers (NPT), number of spikelets per spike (NSP), and number of grains per spike (NGS), the plants were manually recorded from three randomly selected plants and averaged. The 1000 grains weight (TGW) were counted manually and weighed using electrical balance and taken in grams. The plant was harvested and dried to measure the biological yield by converting the data with the recommended plant population per hectare. Grain yield was recorded using electrical balance and multiplied yield per plant with the recommended plant population per hectare to obtain yield per hectare. Harvest Index was taken by the formula;

$$HI = \left( {Grain\;yield/Biological\;yield} \right) \times 100$$

### Statistical analysis

For statistical analysis of data, Fisher’s Analysis of Variance (ANOVA) was used by using Statistix 10.0. Tukey HSD test was used for the comparison of treatment means at a 5% probability level^65^. The heatmap with dendrogram (install.packages("gplots"),, install.packages ("tidyverse"), install.packages ("hclust1d"), install.packages ("pheatmap"), install.packages ("RColorBrewer"), install.packages ("hclust1d"), install.packages ("pheatmap"), install.packages (ggplot2) and Pearson correlation (Package ["corrplot"]) coefficient was constructed using the statistical tool R-studio (v4.3.3), and the graphics were produced using Microsoft Excel (Version, 2016). The partial Eta squared < 0.06 represents an effect size with a small contribution, 0.06 > partial Eta squared < 0.14 represents an effect size with a moderate contribution, and a partial Eta squared > 0.14 represents an effect size with a large contribution^66,67^.

### Plant guidelines

All the experiments were done in compliance with relevant institutional, national, and international guidelines and legislations. High research standards were maintained throughout the experiments and following the various established scientific protocols^68–71^.

## Results

**Figure 1:**
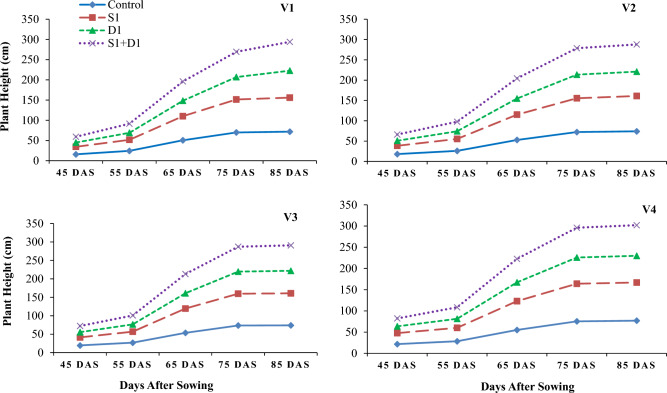
Applications of sulfhydryl thiourea (S_0_ = control-no TU and S_1_ = foliar TU application (500 mg L^−1^) on plant height in wheat varieties (V_1_ = Punjab-2011, V_2_ = Galaxy-2013, V_3_ = Ujala-2016, and V_4_ = Anaaj-2017) under drought stress (D_1_ = control-80% field capacity (FC) and D_2_ = water stress-40% FC).

**Figure 2:**
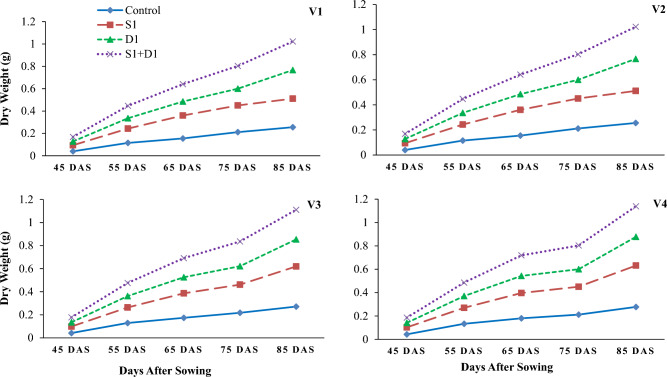
Effects of sulfhydryl thiourea (S_0_ = control-no TU and S_1_ = foliar TU application (500 mg L^−1^) on dry weight in wheat varieties (V_1_ = Punjab-2011, V_2_ = Galaxy-2013, V_3_ = Ujala-2016, and V_4_ = Anaaj-2017) under drought stress (D_1_ = control-80% field capacity (FC) and D_2_ = water stress-40% FC).

**Figure 3:**
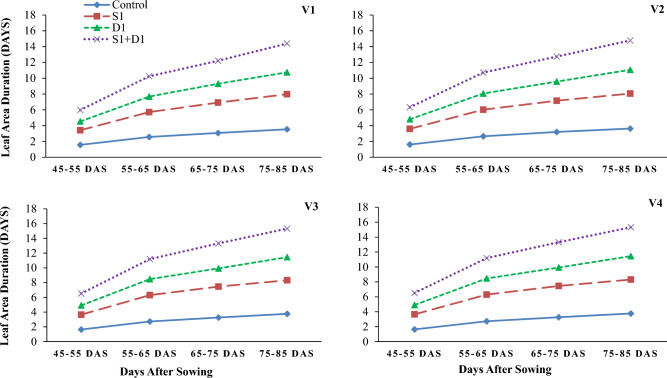
Effects of sulfhydryl thiourea (S_0_ = control-no TU and S_1_ = foliar TU application (500 mg L^−1^) on leaf area duration in wheat varieties (V_1_ = Punjab-2011, V_2_ = Galaxy-2013, V_3_ = Ujala-2016, and V_4_ = Anaaj-2017) under drought stress (D_1_ = control-80% field capacity (FC) and D_2_ = water stress-40% FC).

**Figure 4:**
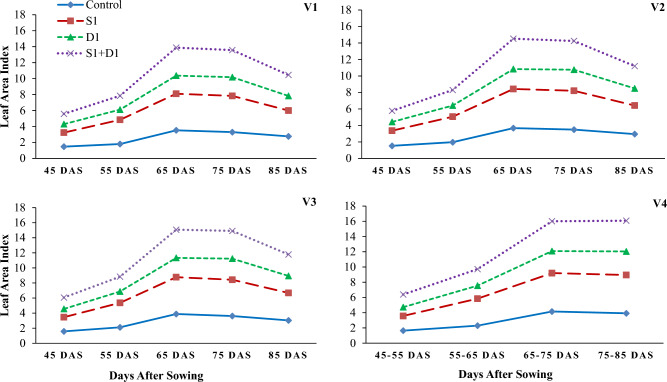
Effects of sulfhydryl thiourea (S_0_ = control-no TU and S_1_ = foliar TU application (500 mg L^−1^) on leaf area index in wheat varieties (V_1_ = Punjab-2011, V_2_ = Galaxy-2013, V_3_ = Ujala-2016, and V_4_ = Anaaj-2017) under drought stress (D_1_ = control-80% field capacity (FC) and D_2_ = water stress-40% FC).

**Figure 5:**
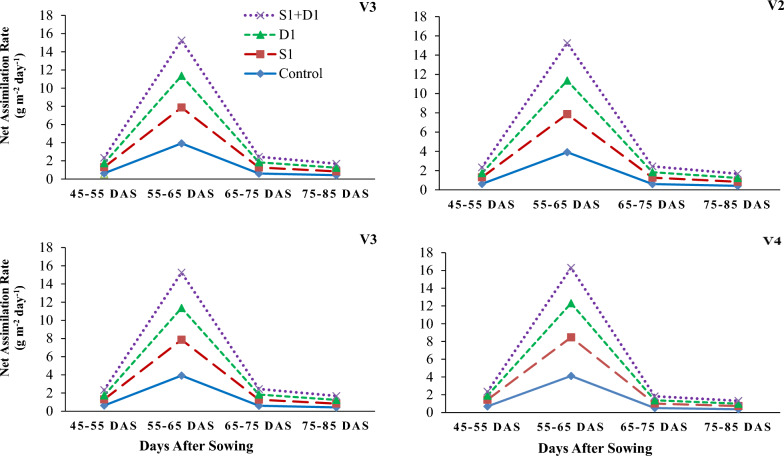
Effects of sulfhydryl thiourea (S_0_ = control-no TU and S_1_ = foliar TU application (500 mg L^−1^) on net assimilation rates in wheat varieties (V_1_ = Punjab-2011, V_2_ = Galaxy-2013, V_3_ = Ujala-2016, and V_4_ = Anaaj-2017) under drought stress (D_1_ = control-80% field capacity (FC) and D_2_ = water stress-40% FC).

**Figure 6:**
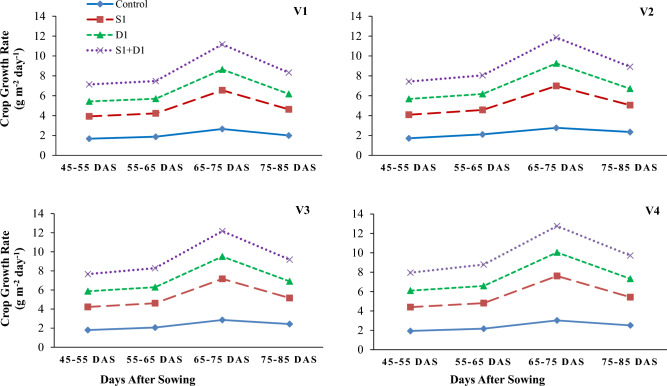
Effects of sulfhydryl thiourea (S_0_ = control-no TU and S_1_ = foliar TU application (500 mg L^−1^) on crop growth rate in wheat varieties (V_1_ = Punjab-2011, V_2_ = Galaxy-2013, V_3_ = Ujala-2016, and V_4_ = Anaaj-2017) under drought stress (D_1_ = control-80% field capacity (FC) and D_2_ = water stress-40% FC).

Drought stress reduced wheat growth, yield, and water relations parameters (Tables 1, 2 and 3; Figs. 1, 2, 3, 4, 5 and 6). Interestingly, foliar thiourea spraying was effective in reducing the adverse effects of drought stress in wheat varieties (Tables 1, 2 and 3; Figs. 1, 2, 3, 4, 5 and 6).

**Table 1:** Effects of thiourea applications on plant physiological parameters in wheat varieties under drought stress.

Drought	Thiourea	Varieties	WP (-MPa)	RWC (%)	PN (μmol CO_2_ m^−2^ s^−1^)	TR (mmol m^−2^ s^−1^)	GS (mol H_2_O m^−2^ s^−1^)	CHLa (mg g^−1^ fw)	CHLb (mg g^−1^ fw)
D0	TU0	V1	0.84 ± 0.002e	52.3 ± 0.23f.	8.85 ± 0.009h	2.58 ± 0.003g	0.171 ± 0.001h	1.03 ± 0.02fg	0.74 ± 0.01fgh
	TU0	V2	0.82 ± 0.002f	54.0 ± 0.39ef	9.21 ± 0.01g	2.67 ± 0.004f.	0.181 ± 0.001g	1.13 ± 0.03ef	0.78 ± 0.01efg
	TU0	V3	0.79 ± 0.001g	55.8 ± 0.38e	9.44 ± 0.01f	2.72 ± 0.001f	0.212 ± 0.000f	1.29 ± 0.02d	0.82 ± 0.01def
	TU0	V4	0.75 ± 0.001hi	58.1 ± 0.20d	10.1 ± 0.00e	2.81 ± 0.07e	0.236 ± 0.001e	1.40 ± 0.03cd	0.92 ± 0.02bcd
	TU1	V1	0.67 ± 0.002k	43.8 ± 0.41j	10.7 ± 0.01d	3.73 ± 0.01d	0.284 ± 0.002d	1.27 ± 0.02de	0.87 ± 0.01cde
	TU1	V2	0.65 ± 0.002l	45.9 ± 0.40i	11.3 ± 0.009c	3.98 ± 0.01c	0.305 ± 0.002c	1.46 ± 0.02bc	0.96 ± 0.01bc
	TU1	V3	0.64 ± 0.001l	47.h ± 0.23i	11.7 ± 0.01b	4.10 ± 0.008b	0.319 ± 0.001b	1.56 ± 0.02b	1.02 ± 0.02ab
	TU1	V4	0.60 ± 0.001d	50.1 ± 0.13g	12.2 ± 0.008a	4.31 ± 0.02a	0.349 ± 0.000a	1.76 ± 0.06a	1.12 ± 0.04a
D1	TU0	V1	1.01 ± 0.0005a	59.7 ± 0.31d	6.10 ± 0.01n	1.96 ± 0.001k	0.093 ± 0.001k	0.76 ± 0.01i	0.49 ± 0.01k
	TU0	V2	0.99 ± 0.001b	62.1 ± 0.42c	6.46 ± 0.006m	2.07 ± 0.005j	0.096 ± 0.003k	0.86 ± 0.01hi	0.55 ± 0.02ik
	TU0	V3	0.97 ± 0.001c	64.5 ± 0.12b	6.79 ± 0.01l	2.28 ± 0.003i	0.097 ± 0.001k	0.95 ± 0.01gh	0.63 ± 0.00ij
	TU0	V4	0.89 ± 0.005d	67.2 ± 0.22a	7.56 ± 0.02k	2.41 ± 0.006h	0.099 ± 0.004k	1.06 ± 0.00fg	0.74 ± 0.01fghi
	TU1	V1	0.78 ± 0.001g	48.4 ± 0.39gh	8.25 ± 0.003j	2.43 ± 0.009h	0.133 ± 0.003j	0.94 ± 0.01gh	0.64 ± 0.00hij
	TU1	V2	0.76 ± 0.001h	50.2 ± 0.86g	8.50 ± 0.008i	2.54 ± 0.01g	0.135 ± 0.001ij	1.01 ± 0.02fg	0.71 ± 0.01ghi
	TU1	V3	0.75 ± 0.002i	52.3 ± 0.10f	8.87 ± 0.009h	2.69 ± 0.005f	0.137 ± 0.001ij	1.12 ± 0.03ef	0.78 ± 0.01efg
	TU1	V4	0.68 ± 0.003j	54.5 ± 0.36e	9.45 ± 0.003f	2.78 ± 0.009e	0.141 ± 0.001i	1.29 ± 0.02d	0.86 ± 0.02cde

V_1_ = Punjab-2011, V_2_ = Galaxy-2013, V_3_ = Ujala-2016, and V_4_ = Anaaj-2017, drought stress; D_0_ = control-85% field capacity (FC) and D_1_ = drought stress-45% FC at vegetative stage, and TU applications; TU_0_ = control-no TU and TU_1_ = foliar TU application @ 500 mg L^−1^. WP = water potential, RWC = relative water content, PN = photosynthetic rate, TR = transpiration rate, GS = stomatal conductance. According to the Tukey HSD test, values for a parameter that has the same case letter do not differ significantly (*p* ≤ *0.05*). Values represent the average of three replicates per treatment ± SE (Standard error). Each parameter was taken twice with an interval of 1 week at the 4th and 5th week after sowing and averaged.

**Table 2:** Effects of thiourea applications on plant biochemical parameters in wheat varieties under drought stress.

Drought	Thiourea	Varieties	Pr (Unit g^−1^ fw)	GB (Unit g^−1^ fw)	TPH (mg g^−1^ fw)	SOD (Unit g^−1^ fw)	POD (Unit g^−1^ fw)	CAT (Unit g^−1^ fw)	MDA (μmol g^−1^ FW)	EL (%)
D0	TU0	V1	1.69 ± 0.02h	18.0 ± 0.57j	3.91 ± 0.0l	26.0 ± 0.57i	35.3 ± 0.088k	35.9 ± 0.44g	5.21 ± 0.12g	18.8 ± 0.12g
	TU0	V2	1.75 ± 0.02h	20.0 ± 0.57ij	4.21 ± 0.08l	28.0 ± 0.58i	41.3 ± 0.33j	36.7 ± 0.57fg	5.06 ± 0.09g	18.2 ± 0.11gh
	TU0	V3	1.86 ± 0.03h	22.1 ± 0.44hi	5.09 ± 0.27k	32.1 ± 0.44h	43.8 ± 0.72ij	40.6 ± 0.33e	4.41 ± 0.08g	17.4 ± 0.05ghi
	TU0	V4	2.05 ± 0.03g	24.6 ± 0.42gh	4.96 ± 0.04k	34.6 ± 0.41gh	49.0 ± 0.28g	45.9 ± 0.57d	3.89 ± 0.05g	17.1 ± 0.13ghi
	TU1	V1	2.12 ± 0.02g	23.0 ± 0.28hi	7.79 ± 0.09i	33.0 ± 0.28h	43.1i ± 0.44j	39.9 ± 0.44ef	2.34 ± 0.04g	14.8 ± 0.08ghi
	TU1	V2	2.20 ± 0.02fg	24.8 ± 0.44gh	8.12 ± 0.05ij	34.6 ± 0.42gh	45.3h ± 0.88i	41.7 ± 0.54e	2.10 ± 0.06g	14.3 ± 0.04ghi
	TU1	V3	2.36 ± 0.03f	26.8 ± 0.41fg	8.66 ± 0.06hi	36.8 ± 0.44fg	47.8 ± 0.92gh	46.6 ± 0.33d	1.99 ± 0.07g	14.0 ± 0.11hi
	TU1	V4	2.58 ± 0.02e	28.5 ± 0.29ef	8.97 ± 0.05gh	38.5 ± 0.28f	50.5 ± 0.50g	51.9 ± 0.52c	1.79 ± 0.04g	13.2 ± 0.10i
D1	TU0	V1	3.34 ± 0.02d	30.8 ± 0.60e	9.55 ± 0.12fg	38.8 ± 0.60f	55.1 ± 0.16f	47.1 ± 0.28d	40.3 ± 0.88a	67.3 ± 1.76a
	TU0	V2	3.51 ± 0.04d	34.1 ± 0.41d	10.2 ± 0.09f	42.1 ± 0.47e	60.5 ± 0.28e	47.5 ± 0.11d	37.3 ± 1.45ab	63.6 ± 0.88ab
	TU0	V3	3.85 ± 0.03c	35.8 ± 0.72d	11.2 ± 0.22e	45.5 ± 0.72d	63.1 ± 0.44de	51.5 ± 0.86c	35.0 ± 1.15bc	60.6 ± 1.45bc
	TU0	V4	4.11 ± 0.06b	39.3 ± 1.48c	12.3 ± 0.03d	49.3 ± 1.48c	68.6 ± 1.09bc	57.1 ± 1.24b	32.6 ± 0.83c	57.0 ± 1.52c
	TU1	V1	3.86 ± 0.01c	40.1 ± 0.60c	13.7 ± 0.12c	50.7 ± 0.56c	66.5 ± 0.50cd	51.1 ± 0.26c	22.6 ± 0.85d	47.6 ± 0.86d
	TU1	V2	4.06 ± 0.03b	42.2 ± 0.40bc	14.4 ± 0.25c	52.2 ± 0.42bc	70.5 ± 0.29b	52.5 ± 0.11c	20.3 ± 0.81de	43.3 ± 0.84de
	TU1	V3	4.22 ± 0.05b	44.1 ± 0.44b	15.3 ± 0.12b	54.1 ± 0.43b	71.5 ± 1.04b	57.5 ± 0.86b	17.80.72ef	40.0 ± 1.15ef
	TU1	V4	4.50 ± 0.03a	47.4 ± 0.29a	16.5 ± 0.21a	57.3 ± 0.29a	76.8 ± 0.41a	63.1 ± 1.22a	14.5 ± 0.28f.	36.1 ± 1.42f

V_1_ = Punjab-2011, V_2_ = Galaxy-2013, V_3_ = Ujala-2016, and V_4_ = A ± naaj-2017, drought stress; D_0_ = control-85% field capacity (FC) and D_1_ = drought stress-45% FC at vegetative stage, and TU applications; TU_0_ = control-no TU and TU_1_ = foliar TU application @ 500 mg L^−1^. CHLa = chlorophyll a, CHLb = chlorophyll b, Pr = proline, GB = glycine betaine, TPH = total phenolics, MDA = malondialdehyde, EL = electrolyte leakage. According to the Tukey HSD test, values for a parameter that has the same case letter do not differ significantly (*p* ≤ *0.05*). Values represent the average of three replicates per treatment ± SE (Standard error). Each parameter was taken twice with an interval of 1 week at the 4th and 5th week after sowing and averaged.

**Table 3:** Effects of thiourea applications on plant yield and yield-related parameters in wheat varieties under drought stress.

Drought	Thiourea	Varieties	NPT	NSS	NGS	TGW (g)	BY (t ha^−1^)	EY (t ha^−1^)	HI
D0	TU0	V1	5.33 ± 0.33bcde	18.5 ± 0.28efg	38 ± 0.57ef	39.0 ± 0.54def	10.0 ± 0.12defg	4.53 ± 0.20fgh	43.5 ± 1.05ef
	TU0	V2	5.33 ± 0.31bcde	19.5 ± 0.29cdef	39 ± 0.88de	40.0 ± 0.57cdef	10.7 ± 0.51cdef	4.89 ± 0.12efg	44.9 ± 0.65def
	TU0	V3	5.66 ± 0.66abcd	20.6 ± 0.44bcde	43 ± 0.58cd	41.3 ± 0.66bcde	11.1 ± 0.56bcde	5.29 ± 0.09def	45.3 ± 0.84cdef
	TU0	V4	6.66 ± 0.31ab	21.8 ± 0.41abc	47 ± 0.53b	42.6 ± 0.88abcd	12.2 ± 0.40abc	5.88 ± 0.06cd	48.2 ± 1.03bcde
	TU1	V1	5.66 ± 0.34abcd	13.8 ± 0.33i	42 ± 0.52cd	41.6 ± 0.88bcde	11.2 ± 0.55bcde	5.93 ± 0.14cd	38.6 ± 1.11f
	TU1	V2	6.00 ± 0.35abc	15.0 ± 0.31hi	44 ± 0.28bc	43.3 ± 0.83abc	12.1 ± 0.18abc	6.52 ± 0.11bc	39.2 ± 0.55f.
	TU1	V3	6.33 ± 0.64abc	16.5 ± 0.44gh	46 ± 0.98b	44.1 ± 0.72ab	12.7 ± 0.14ab	7.01 ± 0.21b	42.7 ± 2.33ef
	TU1	V4	7.66 ± 0.57a	18.0 ± 0.60fg	51 ± 0.58a	45.8 ± 0.92a	13.8 ± 0.41a	8.01 ± 0.10a	45.9 ± 2.42cdef
D1	TU0	V1	3.33 ± 0.66e	20.6 ± 0.45bcde	29 ± 0.53i	31.0 ± 0.57j	8.81 ± 0.06g	3.45 ± 0.14i	49.7 ± 1.43abcde
	TU0	V2	3.66 ± 0.32de	21.6 ± 0.57bc	31 ± 0.57hi	31.8 ± 0.91ij	9.47 ± 0.19fg	3.73 ± 0.26hi	53.7 ± 1.46abc
	TU0	V3	4.33 ± 0.34cde	22.8 ± 0.28ab	33 ± 0.47gh	33.5 ± 0.25hij	9.76 ± 0.14efg	4.16 ± 0.12ghi	55.0 ± 1.87ab
	TU0	V4	4.66 ± 0.63bcde	24.1 ± 0.57a	35 ± 0.58fg	35.0 ± 0.28hi	10.5 ± 0.22cdef	4.85 ± 0.08efg	58.2 ± 1.45a
	TU1	V1	4.33 ± 0.33cde	17.1 ± 0.72fgh	32 ± 0.53gh	35.1 ± 0.60ghi	9.82 ± 0.28efg	4.72 ± 0.18fg	48.2 ± 1.78bcde
	TU1	V2	4.66 ± 0.66bcde	18.6 ± 0.33defg	35 ± 0.57fg	36.4 ± 0.86fgh	10.1 ± 01.8defg	5.18 ± 0.24def	51.1 ± 3.22abcde
	TU1	V3	5.66 ± 0.33abcd	19.5 ± 0.26cdef	37 ± 0.86ef	38.8 ± 0.44efg	11.1 ± 0.17bcde	5.63 ± 0.18cde	53.3 ± 1.77abcd
	TU1	V4	6.33 ± 0.33abc	21.0 ± 0.50bcd	40 ± 0.60de	40.3 ± 0.88cde	11.6 ± 0.25bcd	6.48 ± 0.25bc	55.7 ± 0.72ab

V_1_ = Punjab-2011, V_2_ = Galaxy-2013, V_3_ = Ujala-2016, and V_4_ = Anaaj-2017, drought stress; D_0_ = control-85% field capacity (FC) and D_1_ = drought stress-45% FC at vegetative stage, and TU applications; TU_0_ = control-no TU and TU_1_ = foliar TU application @ 500 mg L^−1^. NPT = number of productive tillers, NSP = number of spikes per plant, NGS = number of grains per spike, TGW = thousand grain weight, BY = biological yield, EY = economical yield, HI = harvest index. According to the Tukey HSD test, values for a parameter that has the same case letter do not differ significantly (*p* ≤ *0.05*). Values represent the average of three replicates per treatment ± SE (Standard error). Each parameter was taken twice with an interval of 1 week at the 4th and 5th week after sowing and averaged.

### Growth parameters

The results revealed that drought stress and thiourea applications significantly (*p* ≤ *0.05*) affected the growth attributes, including PH (Fig. 1), DW (Fig. 2), LAD (Fig. 3), LAI (Fig. 4), CGR (Fig. 5), and NAR (Fig. 6) of different wheat varieties with TU supplementation. Drought stress reduced the PH, DW, LA, LAI, LAD, CGR, and NAR during all the stages when data was recorded periodically. The average percent reduction in growth indices was observed as PH, DW, LAI, LAD, CGR, and NAR were reduced by 17%, 20%, 27%, 21%, 24%, 25%, and 10%, respectively relative to control-no stress. However, plant growth was significantly (*p* ≤ *0.05*) improved with thiourea applications in wheat varieties under drought-stressed conditions. Results have revealed that PH, DW, LAI, LAD, CGR, and NAR were increased by 15%, 20%, 33%, 27%, 26%, and 6%, respectively as compared to control-no TU (Figs. 1, 2, 3, 4, 5 and 6). Among the varieties, the variety Anaaj-2017 outperformed the other wheat varieties in growth indices and was considered a tolerant variety against drought stress; while the sensitive variety was Punjab-2011. Based on the partial Eta squared (η^2^) values, the effects of drought stress were considered the highest factor; followed by thiourea applications and genotypes (varieties) in accordance to the magnitude of effect of drought stress (D) was 0.53; of sulfhydryl thiourea (S), was 0.30; and of variety (V) was 0.06 for plant height. These values indicated that D and S delivered the large contribution (> 0.14) while V was relevant showed some contribution. For the other phenological attributes, the trend of Eta squared was D > S > V for LAI and CGR (Table 4).

**Table 4:** Partial Eta-squared (η^2^) represents the magnitude of the effect.

Source	F	Pr	η^2^	F	Pr	η^2^	F	Pr	η^2^
	Plant height			LAI			CGR		
Variety	8.48	0.0001 ***	0.063	60.8	2.44^−15^***	0.12	3.59	0.021*	0.03
Sulfhydryl thiourea	121.2	5.8^−14^***	0.30	450.6	2.2^−16^***	0.31	102.0	8.2^−13^***	0.33
Drought stress	214.3	2.2^−16^***	0.53	775.8	2.2^−16^***	0.53	151.5	1.6^−15^***	0.49
	RWC			PN			CHL		
Variety	40.7	1.6^−12^***	0.07	272.1	2.2^−16^***	0.06	82.8	2.2^−16^***	0.27
Sulfhydryl thiourea	872.5	2.2^−16^***	0.54	5124.7	2.2^−16^***	0.42	187.5	2.2^−16^***	0.21
Drought stress	562.9	2.2^−16^***	0.35	5124.7	2.2^−16^***	0.50	412.3	2.2^−16^***	0.21
	TGW			SY			BY		
Variety	26.9	7.1^−10^***	0.12	70.9	2.2^−16^***	0.25	33.8	2.7^−11^***	0.31
Sulfhydryl thiourea	121.7	5.4^−14^***	0.19	358.9	2.2^−16^***	0.43	67.5	2.7^−10^***	0.20
Drought stress	383.8	2.2^−16^***	0.61	216.6	2.2^−16^***	0.26	112.4	1.8^−13^***	0.34

LAI = Leaf area index, CGR = Crop growth rate, RWC = Relative water content, PN = Photosynthetic rate, CHL = Chlorophyll contents, TGW = Thousand grain weight, SY = Seed yield, BY = Biological yield.

### Water relation parameters

Thiourea applications delivered significant (*p* ≤ *0.05*) effects on leaf-relative water contents and water potential in wheat varieties grown under drought stress (Table 1). Drought stress negatively influenced the plant water relations traits and reduced the relative water contents and leaf water potential by an average of 17% and 18%, respectively in periodically recorded data as compared to control. Interestingly, leaf water potential and relative water contents were significantly (*p* ≤ *0.05*) improved with thiourea applications in wheat varieties under drought-stressed conditions. The thiourea supplementation increased the relative water contents and leaf water potential by 12% and 22%, respectively relative to control (Table 2). Among the varieties, the Anaaj-2017 showed 8 and 9% more water potential and relative water content as compared to other wheat varieties followed by Ujala-2016 and Galaxy-2013 while, Punjab-2011 showed minimum values of water potential and relative water content. Based on the partial Eta squared (η^2^) values, the effect of drought stress was highest followed by thiourea applications and genotypes as the magnitude of effect of drought stress (D) was 0.35, of sulfhydryl thiourea (S), was 0.54, and of variety (V) was 0.07 for relative water contents, which suggested that D and S showed the large contribution (> 0.14) While V showed medium contribution (Table 4).

### Gas exchange parameters

Thiourea applications delivered significant (*p* ≤ *0.05*) effects on *PN*, *TR*, and *GS* in wheat varieties grown under drought stress (Table 1). Drought stress negatively influenced the plant gas exchange parameters and reduced the *PN*, *TR*, and *GS* by 26%, 29%, and 55% respectively as compared to control-no water stress. On the other hand, the thiourea applications positively influenced the gas exchange parameters in wheat varieties under drought stress. The thiourea supplementation minimized the drought stress impact by increasing the *PN*, *TR*, and *GS* by 26%, 37%, and 52% respectively as compared to control (Table 1). Among the varieties, the Anaaj-2017 showed more *PN*, *TR*, and *GS* by 11%, 9%, and 14% as compared to other wheat varieties followed by Ujala-2016 and Galaxy-2013 while, Punjab-2011 showed minimum values of *PN*, *TR*, and *GS*. Based on the partial Eta squared (η^2^) values, the effect of drought stress was highest followed by thiourea applications and genotypes as the magnitude of effect of drought stress (D) was 0.50, of sulfhydryl thiourea (S), was 0.42, and of variety (V) was 0.06 for photosynthetic rate, which suggested that D and S showed the large contribution (> 0.14) While V showed medium contribution. For other gas exchange attributes, the trend of Eta squared was D > S > V for stomatal conductance (Table 4).

### Chlorophyll contents

Drought stress and thiourea applications significantly (*p* ≤ *0.05*) affected the chlorophyll content in wheat varieties (Table 2). Drought stress negatively influenced the foliar chlorophyll content and reduced the CHLa and CHLb by 26% and 25% respectively as compared to control-no drought stress. On the other hand, the thiourea applications improved the foliar chlorophyll content under drought stress. The thiourea supplementation improved the CHLa and CHLb by 23% and 22%, respectively as compared to the control (Table 2). Among the varieties, the Anaaj-2017 outperformed other wheat varieties in relation to chlorophyll content and showed 24% and 21% more CHLa and CHLb as compared to other varieties and stood as a tolerant variety against drought stress while the sensitive variety was Punjab-2011. Based on the partial Eta squared (η^2^) values, the effect of drought stress was highest followed by thiourea applications and genotypes as the magnitude of effect of drought stress (D) was 0.21, of sulfhydryl thiourea (S), was 0.21, and of variety (V) was 0.27 for chlorophyll contents, which suggested that D, V, and S showed the large contribution (> 0.14) (Table 4).

### Malondialdehyde and electrolyte leakage

The stress indicators were significantly (*p* ≤ *0.05*) affected under drought stress and thiourea applications in four wheat varieties (Table 2). Drought stress negatively influenced wheat growth by increasing the accumulation of MDA and EL by 722% and 224% respectively as compared to control-no drought stress. However, the thiourea application improved the plant defense system and reduced the production of MDA and EL by 49% and 30%, respectively as compared to control (Table 2). Among the varieties, the Punjab-2011 showed more MDA and EL by 18% and 12% as compared to other wheat varieties followed by Galaxy-2013 and Ujala-2016 while Anaaj-2017 showed minimum values of MDA and EL. Partial Eta squared (η^2^) values showed the maximum effect was shown by drought stress as compared to thiourea applications and genotypes, respectively as the magnitude of the effect of drought stress (D) was 0.76, sulfhydryl thiourea (S) was 0.13, and variety (V) was 0.01 for plant height, which suggested that D showed the large contribution (> 0.14), S showed the medium contribution (< 0.14), While V showed small contribution (< 0.06). For other phenological attributes, the trend of Eta squared was D > S > V for EL (Table 5).

**Table 5:** Partial Eta-squared (η^2^) represents the magnitude of the effect.

Source	F	Pr	η^2^	F	Pr	η^2^	F	Pr	η^2^
	Pr			GB			Tph		
Variety	91.7	2.1^−16^**	0.04	50.7	5.0^−14^***	0.08	53.1	2.3^−14^***	0.03
Sulfhydryl thiourea	341.3	2.2^−16^***	0.05	245.3	2.2^−16^***	0.13	1137.3	2.2^−16^***	0.26
Drought stress	5384.3	2.1^−16^***	0.89	1435.0	2.2^−16^***	0.76	2961.0	2.2^−16^***	0.68
	SOD			POD			CAT		
Variety	57.5	6.2^−15^***	0.10	66.6	5.2^e−16^***	0.10	258.6	2.2^−16^***	0.32
Sulfhydryl thiourea	268.8	2.2^−16^***	0.16	146.0	2.9^−15^***	0.07	283.1	2.2^−16^***	0.12
Drought stress	1181.9	2.2^−16^***	0.70	1494.7	2.2^−16^***	0.79	1251.5	2.2^−16^***	0.53
	GS			MDA			EL		
Variety	5.12	0.004**	0.02	2.28	0.09	0.01	3.45	0.02*	0.01
Sulfhydryl thiourea	222.8	2.2^−16^***	0.32	64.2	5.3^e−10^***	0.13	70.6	1.5^−10^***	0.09
Drought stress	412.2	2.2^−16^***	0.59	373.8	2.2^−16^***	0.76	624.8	2.2^−16^***	0.83

Pr = Proline, GB = Glycine betaine, Tph = Total phenolics, SOD = Superoxide dismutase, POD = Peroxidase, CAT = Catalase, GS = Stomatal conductance, MDA = Malondialdehyde, EL = Electrolyte leakage.

### Antioxidants

Drought stress and thiourea applications significantly (*p* ≤ *0.05*) affected the superoxide dismutase (SOD), peroxidase (POD), and catalase (CAT) in wheat varieties (Table 2). Drought stress significantly affected the production of SOD, POD, and CAT in wheat varieties as compared to control-no drought stress. On the other hand, the thiourea applications ameliorated the deleterious impact of drought stress by improving the production and activities of SOD, POD, and CAT under drought stress. The thiourea supplementation improved the SOD, POD, and CAT by 20%, 13%, and 12%, respectively as compared to control (Table 2). Among the wheat varieties, the Anaaj-2017 showed 14%, 14%, and 19% more SOD, POD, and CAT as compared to other wheat varieties followed by Ujala-2016 and Galaxy-2013 while, Punjab-2011 showed minimum values of SOD, POD, and CAT. Drought stress showed the maximum effect according to η^2^ which was followed by sulfhydryl thiourea and crop varieties as the magnitude of effect 0.70 for D, 0.16 for S, and 0.10 for V for SOD, which suggested that D and S showed a large contribution (> 0.14) While V showed medium contribution (< 0.14). For other antioxidants, the trend of Eta squared was D > V > S for POD and CAT (Table 5).

### Osmolyte/Metabolite production

Drought stress and thiourea applications significantly (*p* ≤ *0.05*) affected the proline (Pr), glycine betaine (GB), and total phenolics (TPH) in wheat varieties (Table 2). Drought stress significantly affected the production of Pr, GB, and TPH in wheat varieties as compared to control-no drought stress. On the other hand, the thiourea applications ameliorated the deleterious impact of drought stress by improving the production and activities of Pr, GB, and TPH under drought stress. The thiourea supplementation improved the Pr, GB, and TPH by 17%, 23%, and 52%, respectively as compared to control-no thiourea (Table 2). Among the wheat varieties, the Anaaj-2017 showed 14%, 16%, and 14% more Pr, GB, and TPH as compared to other wheat varieties followed by Ujala-2016 and Galaxy-2013 while, Punjab-2011 showed minimum values of Pr, GB, and TPH. Based on the partial Eta squared (η^2^) values, the effect of drought stress was highest followed by thiourea applications and genotypes as the magnitude of effect of drought stress (D) was 0.70, sulfhydryl thiourea (S) was 0.16, and of variety (V) was 0.10 for P, which suggested that D and S showed the large contribution (> 0.14), While V showed medium contribution (< 0.14). For other metabolites, the trend of Eta squared was D > V > S for GB and Tph (Table 5).

### Yield attributes

Thiourea applications showed a significant (*p* ≤ *0.05*) effect on yield and yield-related parameters in wheat varieties grown under drought stress (Table 3). Drought stress reduced the number of productive tillers (NPT), number of spikelets per spike (NSS), number of grains per spike (NGS), thousand-grain weight (TGW), biological yield (BY), economical yield (EY), and harvest index (HI) by 24%, 18%, 22%, 17%, 14%, 21%, and 6%, respectively as compared to control-no drought stress. In contrast, the application of thiourea improved the NPT, NSS, NGS, TGW, BY, EY, and HI by 20%, 19%, 12%, 11%, 12%, 34%, and 22% respectively as compared to control-no thiourea. A significant (*p* ≤ *0.05*) difference was found among varieties of grain yield and yield-related parameters (Table 3). Annaj-2017 performed best in terms of yield parameters and showed 26%, 14%, 15%, 8%, 14%, 24%, and 10% more NPT, NSS, NGS, TGW, BY, EY, and HI as compared to other varieties. Based on the partial Eta squared (η^2^) values, the effect of drought stress was highest followed by thiourea applications and genotypes as the magnitude of effect of drought stress (D) was 0.61, sulfhydryl thiourea (S) was 0.19, and of variety (V) was 0.12 for TGW, which suggested that D and S showed the large contribution (> 0.14) While V showed medium contribution (< 0.14). For other phenological attributes, the trend of Eta squared was D > S > V for LAI and CGR (Table 5).

### Correlation matrix

**Figure 7:**
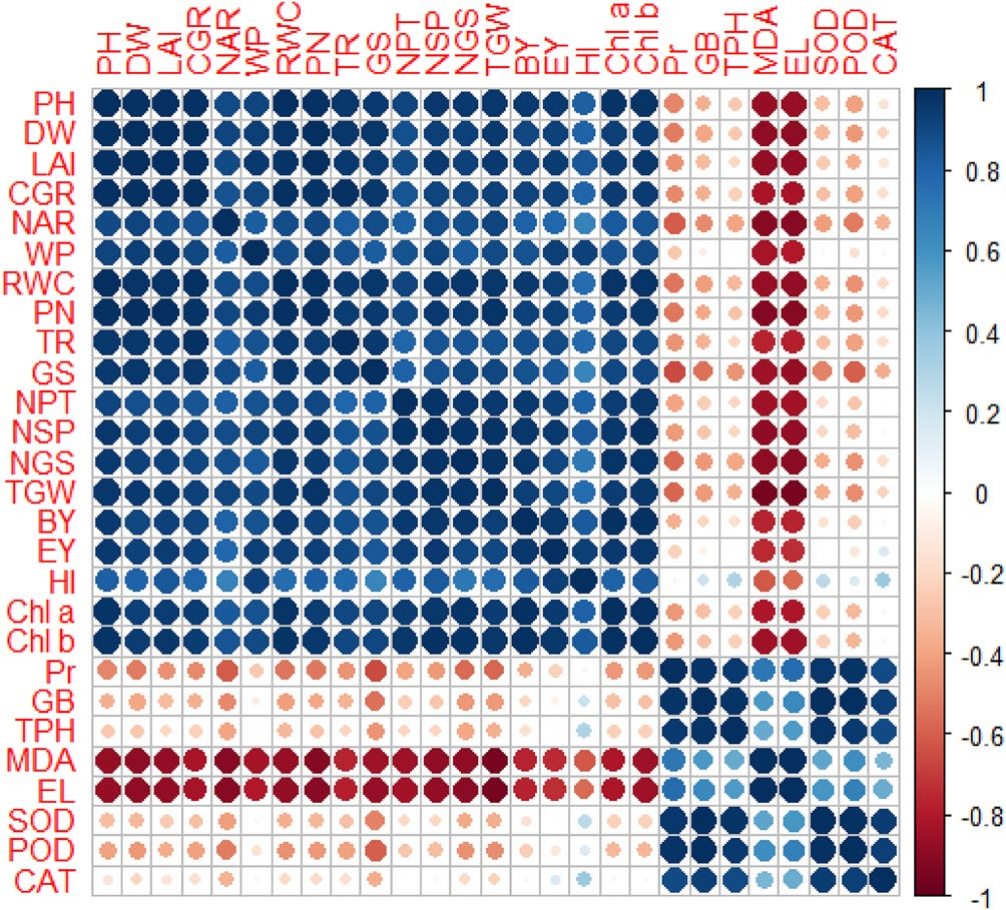
Correlation matrix for the effects of thiourea applications (S_1_ = control-no thiourea and S_2_ = foliar thiourea application @ 500 mg L^−1^ at 30 days after sowing) on plant phenological, morphological, physiological, and biochemical attributes on wheat varieties (V_1_ = Punjab-2011, V_2_ = Galaxy-2013, V_3_ = Ujala-2016, and V_4_ = Anaaj-2017) under drought stress (D_1_ = control-80% field capacity (FC) and D_2_ = water stress-40% FC). Plant height (PH), dry weight (DW), leaf area index (LAI), crop growth rate (CGR), net assimilation rate (NAR), water potential (WP), relative water content (RWC), photosynthetic rate (PN), transpiration rate (TR), stomatal conductance (GS), number of productive tillers (NPT), number of spikelets per spike (NSS), number of grains per spike (NGS), thousand grain weight (TGW), biological yield (BY), economical yield (EY), and harvest index (HI), proline (Pr), glycine betaine (GB), total phenolics (TPH), malondialdehyde (MDA), electrolyte leakage (EL), SUPEROXIDE DISMUTASE (SOD), peroxidase (POD), catalase (CAT).

**Figure 8:**
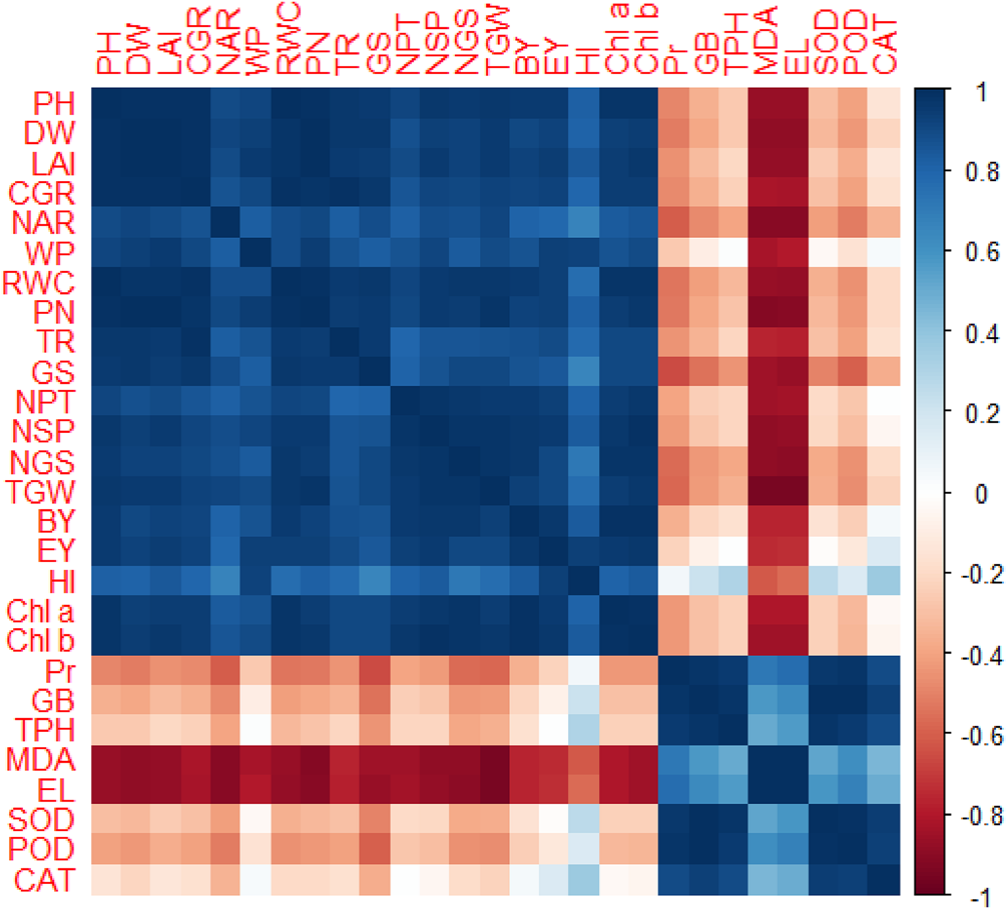
Correlation matrix for the effect of thiourea applications (S_1_ = control-no thiourea and S_2_ = foliar thiourea application @ 500 mg L^−1^ at 30 days after sowing) on plant phenological, morphological, physiological, and biochemical attributes on wheat varieties (V_1_ = Punjab-2011, V_2_ = Galaxy-2013, V_3_ = Ujala-2016, and V_4_ = Anaaj-2017) under drought stress (D_1_ = control-80% field capacity (FC) and D_2_ = water stress-40% FC). Plant height (PH), dry weight (DW), leaf area index (LAI), crop growth rate (CGR), net assimilation rate (NAR), water potential (WP), relative water content (RWC), photosynthetic rate (PN), transpiration rate (TR), stomatal conductance (GS), number of productive tillers (NPT), number of spikelets per spike (NSS), number of grains per spike (NGS), thousand grain weight (TGW), biological yield (BY), economical yield (EY), and harvest index (HI), proline (Pr), glycine betaine (GB), total phenolics (TPH), malondialdehyde (MDA), electrolyte leakage (EL), SUPEROXIDE DISMUTASE (SOD), peroxidase (POD), catalase (CAT).

The correlation matrix represents the correlation among different parameters of wheat varieties grown under drought stress and thiourea applications (Figs. 7 and 8). Plant phenological attributes have shown a strong positive correlation with plant physiological and biochemical attributes. Plant height, dry weight, and seed yield showed a strong positive correlation with plant water status and photosynthetic rate. In addition, the increased leaf area and chlorophyll contents showed a positive correlation with the crop growth rate and net assimilation rate which was also positively correlated with photosynthetic rate. Among the physiological attributes, stomatal conductance and transpiration rate showed a strong positive correlation with plant water status including water potential and relative water content which is also associated with the photosynthetic rate and crop growth and development. Plant physiological and penological attributes were strongly correlated with the crop yield and yield-related parameters in wheat varieties. Among the biochemical parameters, antioxidant content showed a negative correlation with the plant's physiological and yield parameters as plants paid the price by diverting photosynthates to produce more antioxidants and osmolytes. In addition, stress indicators have shown a strong negative correlation with the phonological, physiological, and yield parameters in all wheat varieties. In crux, plant growth and yield showed a strong negative correlation with MDA and EL revealing that.

### Heatmap

**Figure 9:**
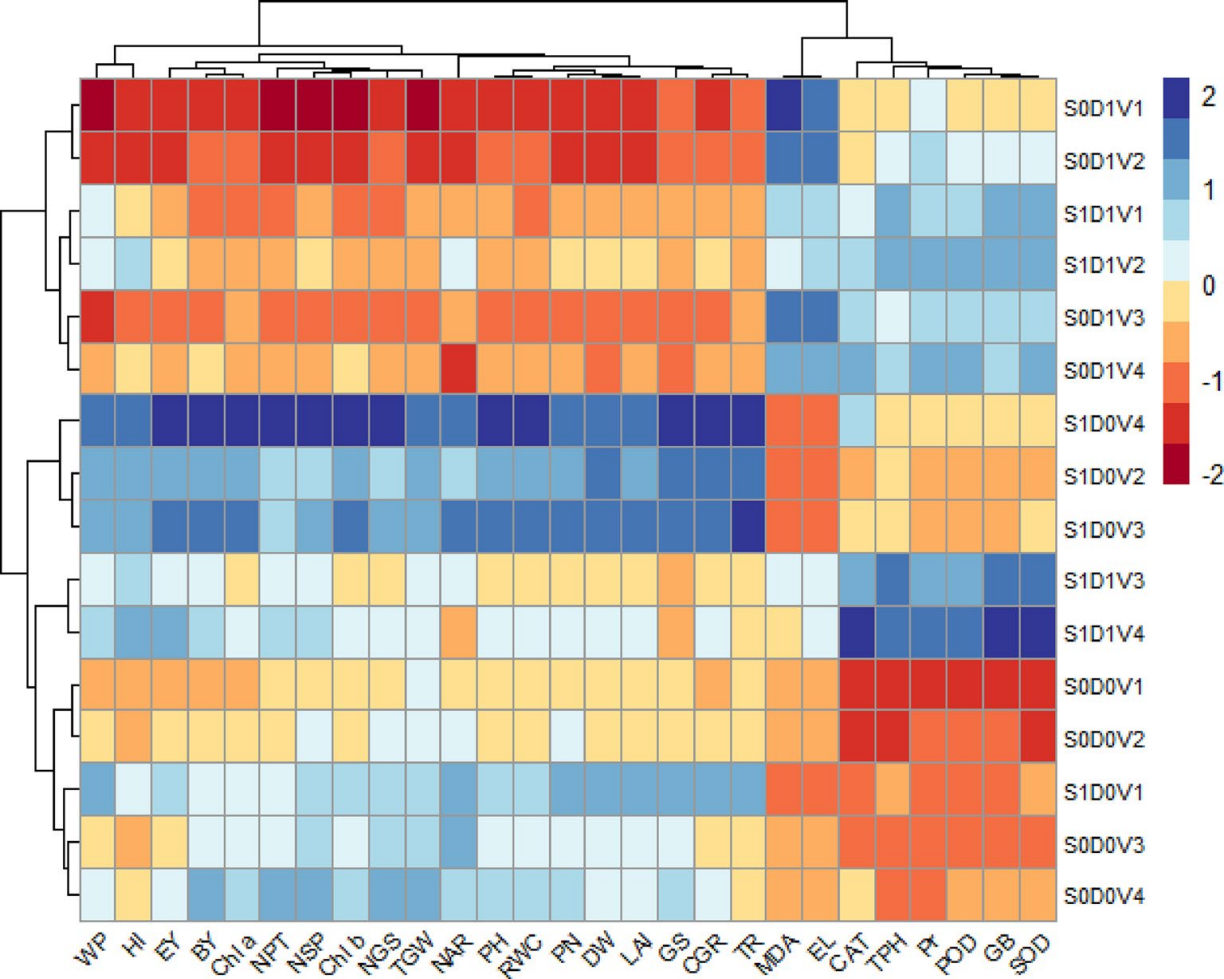
The clustered heatmap of a number of physiological, biochemical, and quality attributes, including Plant height (PH), dry weight (DW), leaf area index (LAI), crop growth rate (CGR), net assimilation rate (NAR), water potential (WP), relative water content (RWC), photosynthetic rate (PN), transpiration rate (TR), stomatal conductance (GS), number of productive tillers (NPT), number of spikelets per spike (NSS), number of wheat grains per spike (NGS), thousand grain weight (TGW), biological yield (BY), economical yield (EY), and harvest index (HI), proline (Pr), glycine betaine (GB), total phenolics (TPH), malondialdehyde (MDA), electrolyte leakage (EL), SUPEROXIDE DISMUTASE (SOD), peroxidase (POD), catalase (CAT) of wheat varieties grown under drought stress.

To observe the effects of thiourea on various parameters in the wheat varieties under drought stress conditions, a two-way clustered heatmap was drawn. The measurements were categorized based on how comparable they were at the various stages of treatment, and colored squares represented the relationship between them. The maroon color shows strongly and red shows slightly positive while blue and light blue shows the negative correlation of different parameters impacted by thiourea under drought stress conditions. Heatmap has clustered into four groups. In the first group, organic osmolytes and antioxidants (transpiration rate (TR), proline (Pr), glycine betaine (GB), total phenolics (TPH), malondialdehyde (MDA), electrolyte leakage (EL), superoxide dismutase (SOD), peroxidase (POD) and catalase (CAT) was clustered. Malondialdehyde (MDA), and electrolyte leakage (EL), are strongly positively correlated to control in V_3_ = Ujala-2016, and V_4_ = Anaaj-2017 and weakly correlated in V_1_ = Punjab-2011, V_2_ = Galaxy-2013, at thiourea 500 mg/L under drought stress. While without drought stress conditions, (transpiration rate (TR), proline (Pr), glycine betaine (GB), total phenolics (TPH), SUPEROXIDE DISMUTASE (SOD), peroxidase (POD) and catalase (CAT) showed strong negative correlation in V_3_ = Ujala-2016, and V_4_ = Anaaj-2017 and weakly correlated in V_1_ = Punjab-2011, V_2_ = Galaxy-2013 at thiourea 500 mg/L. Showing that the application of thiourea 500 mg/L helped in decreasing drought stress by increasing organic osmolyte content and antioxidants for mitigating adverse effects of oxidative damage. Among varieties V_3_ = Ujala-2016, and V_4_ = Anaaj-2017 showed more drought stress tolerance as compared to V_1_ = Punjab-2011, and V_2_ = Galaxy-2013. The second group is the largest group containing growth attributes (Plant height (PH), dry weight (DW), leaf area index (LAI), crop growth rate (CGR), water relation attributes net assimilation rate (NAR), water potential (WP), relative water content (RWC), gas exchange attributes (photosynthetic rate (PN), transpiration rate (TR), stomatal conductance (GS), and yield attributes (number of productive tillers (NPT), number of spikelets per spike (NSS), number of grains per spike (NGS), thousand-grain weight (TGW), biological yield (BY), economical yield (EY), and harvest index (HI). All these parameters are strongly positively correlated to control at thiourea 500 mg/L application level in V_3_ = Ujala-2016, and V_4_ = Anaaj-2017 and weakly correlated in V_1_ = Punjab-2011, V_2_ = Galaxy-2013 without drought stress. However, under drought stress conditions, V_3_ = Ujala-2016, and V_4_ = Anaaj-2017 showed weak positive and V_1_ = Punjab-2011, and V_2_ = Galaxy-2013 showed negative correlation without the application of thiourea. These findings showed that the application of thiourea 500 mg/L proved beneficial in increasing growth attributes, photosynthetic contents, gas exchange, water relations, and yield attributes of all wheat varieties. (Fig. 9).

## Discussion

One significant environmental factor that can affect the plant morph-physiology, biochemistry, and yield attributes in wheat is drought stress particularly in rainfed arid and semi-arid regions^72^. Under drought stress, however, thiourea treatments significantly restored seedling development leading to better crop performance by detoxifying ROS and nutrient translocation^73^. The results demonstrated that thiourea application upregulated the plant defense system that helped the wheat plants to maintain proper gaseous exchange and plant water status during the drought episode (Tables 2, 3, 4 and 5). Significant differences were observed between wheat varieties under drought stress in terms of plant morphophysiological characteristics that can be used in wheat improvement programs intended to create varieties resistant to drought stress.

Drought stress reduces the growth and development of crops due to the possible effects of oxidative stress to disrupt the plant antioxidant system^25,74^. Results have revealed that drought stress negatively affected the development of wheat plants due to a significant reduction in plant water status. All the growth and phenological attributes, including PH, DW, LAI, LAD, CGR, and NAR were significantly reduced under drought stress in wheat varieties; however, the wheat variety (Anaaj-2017) had growth parameters than the control among the rest of the varieties (Figs. 2, 3, 4, 5, 6 and 7). The genotypes showing better CGR, LAI, and NAR under drought stress, at some level, can better tolerate the limited water supply owing to sustained dry matter accumulation and growth as has been seen in this study. Leaf area reduction is usually the first observable effect of drought stress in crop plants;  [Figs. 2, 3, 4, 5, 6 and 7]^75–77^. The reduction in growth and phenology may be attributed to the reduction in plant tissue water ontent and nutrient uptake by the roots^78^. Interestingly, the exogenous thiourea foliar spray restored plant growth and phenological attributes in wheat varieties grown under normal and drought-stressed conditions; and more distinct improvement was observed in wheat variety Anaaj-2017 as compared to other varieties. A positive correlation between plant height and dry weight was observed with the LAI, LAD, CGR, and NAR which are also clustered together (Figs. 7, 8 and 9). Following thiourea foliar spray, the improvement in plant phenology may be attributed to the higher photosynthetic rates or endogenous phytohormonal changes in plants under drought stress^25^. Better plant development under thiourea treatments may also be linked to improved tissue water status, according to Upadhyay et al.^45^, because of the improved relative water content in stressful environments. All things considered; our research demonstrated the significance of thiourea treatments in enhancing wheat seedling growth during drought stress. Overall increases in wheat growth indices under drought stress show that the investigated wheat varieties had high levels of antioxidant enzyme activity and high carboxylation efficiency^76,75^. Supplementing with thiourea enhances photosynthesis and the translocation of these photosynthates in plants, which reduces the susceptibility of cereals, pulses, oilseeds, and spices to salinity and drought^79,80^. Thiourea supplementations to increase the flag leaf area or photosynthetic tissues as well as modifying the wheat characteristics by increasing the potential of antioxidants, the integrity of the membrane, and the components of yield^81^. The correlation matrix showed that plant growth parameters had a strong positive correlation with plant physiological parameters including water potential and photosynthetic rates. However, all the growth parameters were strongly positively correlated showing that the change in one parameter may affect the performance of other parameters with the same intensity.

Drought stress reduced the physiological attributes in wheat varieties such as photosynthetic rates, transpiration, and stomatal conductances^25,35^. The correlation matrix showed that causing a reduction in LAI, CGR, and NAR (Table 1; Figs. 3, 4, 5 and 6). The reduction of leaf area under drought stress of wheat plants is directly related to photosynthesis and transpiration strength^73^. To maintain their water potential, plants limit gas exchange by adjusting their stomatal apertures to minimize the rate of transpiration^82^. Nonetheless, reduced photosynthesis caused by a drop in intercellular CO_2_ concentration (Ci) harms the growth and development of plants^24^. However, the thiourea application enhanced the plant gas exchange parameters under drought stress in wheat varieties and more response among wheat varieties was observed in Anaaj-2017 (Table 1). Thiourea application improves the rates of photosynthesis and assimilation of photosynthates which helps to increase crop growth and development^79,83^. Plants rely heavily on transpirational cooling action to lower leaf temperature since temperature is essential to the chemical and biological activities that occur inside leaves when water is scarce. Thiourea treatment also enhances gas exchange characteristics, improving transpiration rate, boosting photosynthetic efficiency, and assisting in the fight against abiotic stressors^81^.

Reduced water availability alters plant metabolism, which lowers cell turgidity and water balance and adversely reducing growth^84^. Our findings showed that drought stress negatively impacted the physiology of plants by lowering their relative water levels and leaf water potential, which in turn affected the rates of photosynthesis and stomatal conductances (Table 1, Figs. 2, 3, 4, 5, 6 and 7). One crucial adaptive approach used by plant species to withstand drought stress is maintaining the water content of their tissues, which enables the plants to continue growing under drought^8,24,25,85^. Results have revealed that thiourea applications improved the water relation parameters in all wheat varieties and more improvement was observed in Anaaj-2017 under drought stress. Based on our observations and from literature, it is plausible that thiourea applications improve the relative water content; that in turn maintain the stomatal conductances, transpiration, and photosynthetic ompetence during the abiotic stress periods^81^. Among the physiological attributes, the correlation matrix showed that the wheat plant water relations showed a strong positive correlation with the gas exchange attributes as the improvement in plant water status showed a direct linkage with the increase in photosynthetic rate.

The most significant pigment involved in photosynthetic processes is chlorophyll, which regulates plant photosynthetic capability by absorbing light energy^86,87^. The results of this study showed that the chlorophyll contents in wheat varieties were significantly reduced under drought stress compared to the control. The maximum reduction of chlorophyll content was observed in the wheat variety Punjab-2011 compared to other varieties, while the minimum reduction was observed in Anaaj-2017 (Table 1). The reduction in chlorophyll is linked to the stressed-induced damage to photosynthetic machinery, protein instability, and activities of chlorophyllase-enzyme. Our results found that foliar application of thiourea improved chlorophyll contents in wheat seedlings under drought stress. However, the maximum improvement of chlorophyll content was observed in the wheat variety Anaaj-2017 compared to other varieties under normal and drought stress (Table 1). Thiourea supplementation led to improved photosynthetic pigments and carbon fixation in plants due to the improved plant water status^80^, reduced electrolyte leakage^41^, higher proline content^88^, and higher nutrient uptake^89^. These high chlorophyll values, under drought, were indicative of physiological adaptations to cope with high irradiance and avoiding light-induced oxidative damage^90,91^. A positive correlation between water relations, gas exchange attributes, and chlorophyll contents was observed (Figs. 7 and 9) under thiourea applications may suggest the role of thiourea as a plant growth regulator. The results have demonstrated a highly positive correlation between seed yield and yield attributes in wheat varieties under drought stress.

Plants possess defense mechanisms to deal with lipid peroxidation and ROS production, such as antioxidants and osmolytes accumulation^92^. Thiourea supplementation enhanced wheat seedlings' resistance to drought-induced osmotic stress by raising SOD and POD activities^93^. Results have revealed that drought stress-induced oxidative stress increased the production of MDA and EL in-wheat varieties, while, maximum damage to these stress indicators was observed in Punjab-2011, and minimum damage was observed in Anaaj-201 which was the stand-out variety to tolerate drought stress (Table 2). However, thiourea applications showed an alleviative role in wheat varieties against drought-induced oxidative damage by improving the activities of antioxidants, including SOD, POD, and CAT. Among the wheat varieties, Anaaj-2017 showed maximum improvement in antioxidant activities as compared to other varieties and reduced the concentration of MDA and EL. Superoxide dismutase assists in the detoxification of superoxide (O_2_^−^), which can reduce the damage caused by ROS during dry circumstances. Accordingly, thiourea concentrations increase seedling development and drought stress tolerance in all wheat varieties when administered as a foliar spray. The initial line of defence against high ROS concentration in plants is provided by superoxide dismutase. Superoxide dismutase decreases the risk of •OH production by converting O_2_• − to H_2_O_2_^47^. Several enzymes, including peroxidases and CAT, make sure that H_2_O_2_ is removed from plant cells^94^. Catalase effectively contributes to the removal of H_2_O_2_ by transforming it into molecular oxygen. The current investigation found that CAT activity increased in response to drought stress and thiourea treatments, which could be the reason for the decrease in EL and MDA levels. Peroxidases function well even at low H_2_O_2_ concentrations^95^. In this study, wheat seedlings under drought stress showed a notable increase in proline, glycine betaine, and total phenolics. According to Ahmad et al.^96^, phenolic compounds have antioxidant qualities to get rid of ROS since they are agents that donate electrons. Results have revealed that drought stress-induced oxidative stress was detoxified by the osmoregulation balance in wheat varieties, while, maximum improvement was observed in Anaaj-2017 and minimum damage was observed in Punjab-2011 which was the stand-out variety to tolerate drought stress (Table 2). However, thiourea applications ameliorated the negative impact by improving osmoregulation in wheat varieties against drought-induced oxidative damages by improving the activities of Pr, GB, and Tph (Table 3). Among the wheat varieties, Anaaj-2017 showed maximum improvement in osmolyte production as compared to other varieties. Higher concentrations of proline and phenolics were found in leaves from sources under water stress, which may have shielded the progeny plants' membranes from ROS damage. Additionally, progeny plants that demonstrated their protective activity against ROS showed increased de novo synthesis of osmolytes, which decreased MDA accumulation owing to lipid peroxidation^97^. According to Ullah et al.^76^, wheat genotypes with higher levels of compatible and metabolomic solutes are more resistant to drought than genotypes with lower accumulation of compatible solutes. Apart from its function in osmotic adjustment, proline also scavenges ROS and shields plant cell membranes and proteins from oxidative damage caused by dehydration^98^. The results have shown that a positive correlation between proline content and anti-oxidative activity and soluble sugars and proteins, as well as a negative relationship with malondialdehyde and electrolyte leakage (Figs. 7 and 9), may suggest an ameliorative role for proline and glycine betaine (moreover its key role as an osmolyte). The results have demonstrated a high correlation between the activity of antioxidant enzymes and drought stress resistance in relation to wheat varieties. The upregulation in the activities of the ascorbate peroxidase, catalase, and protease under drought in wheat varieties was significant. Also, a positive relationship between seed yield and antioxidant activities was observed, and the authors stated that this might be used to identify tolerance varieties under high drought stress. The correlation matrix showed that the production of oxidants and antioxidants showed a negative correlation with plant morphophysiological parameters. Antioxidant contents showed a moderate negative correlation with plant morphophysiological parameters as the plant sacrificed its growth and development to improve antioxidant content to improve its tolerance against the overproduction of antioxidants.

According to Waraich et al.^49^, drought stress negatively impacts plant growth and yield because it reduces growth and yield-related parameters due to water limitations at any stage of growth (Table 3). The findings highlighted that drought stress had a substantial impact on wheat output when compared to the control group that did not experience any stress. The number of grains per spike and the 1000-grain weight decreased, which led to a fall in crop yield (Table 3). Our observations were in agreement with the findings of Lin et al.^99^ who reported lesser grains per spike for plants under terminal drought conditions. Previous researchers had reported that drought stress has a detrimental effect on growth and crop yield, with the degree of the drought stress and the stage of plant growth determining how much the yield decreases^72,100^. Following photosynthesis, the partitioning of photoassimilates and its accumulation  has a major role in determining grain yield^72,101^. However, thiourea applications improved the yield and yield-related attributes in wheat varieties under drought stress, and maximum reduction was observed in Punjab-2011, and minimum reduction was noted in Anaaj-2017. The thiourea applications enhanced the wheat grain yield by the improvement in plant water status which caused the enhancement of yield attributes including 1000-grain weight and number of productive tillers (Table 1 and 3). Further, the higher number of grains per spike and 1000-grain weight leads to a high grain yield^102,103^. Thiourea increased grain weight due to efficient photosynthates translocation as well as balancing the source-to-sink relationship^43,81^. This also relates to the finding of Kumar et al.^103^ that in wheat, foliar application of thiourea (500 ppm) modifies the biochemical composition, productive tillers, and leaf biomass.

Our results and other observations from literature demonstrated that exogenously applied thiourea can  ameliorate the negative effects on plant growth and devlopment brought upon by abiotic stressors like extreme temperatures, drought, and heavy metal-metalloid pollution^39,41,42,45,46,79, 80,93^. This opens up the possibilty of using thiourea as an effective and affordable supplement to increase agricultural output and resilience in the face of difficult and unpredictable environmental conditions brought on by climate change.

## Conclusion

The use of thiourea foliar applications delivered significant effects on wheat performance under both drought-stressed and non-stressed conditions. Thiourea applications to wheat plants altered growth indices and improved physiological attributes including water content and photosynthetic rates. Specifically, thiourea foliar applications restored wheat growth during drought stress by improving the antioxidant defense system and osmolyte regulations; concomitantly reducing the MDA production and electrolyte leakage. Interestingly, the variety Anaaj-2017 demonstrated better resilience against drought stress through the thiourea-induced defense system as compared to other wheat varieties.

## Permissions

Permissions were obtained to use the seeds for research purposes.

### Supplementary Information

Supplementary Information.

## Data Availability

All data generated or analyzed during this study are included in this published article.
