# Supplementary material for: Mitigating drought-induced oxidative stress in wheat (Triticum aestivum L.) through foliar application of sulfhydryl thiourea
Source: Sci Rep. 2024 Jul 10;14:15985. doi: 10.1038/s41598-024-66506-y (PMC11237047; doi:10.1038/s41598-024-66506-y)
Supplement: Supplementary file 1 — Supplementary Information. [file 41598_2024_66506_MOESM1_ESM.docx]

**P values for the phenological, physiological, biochemical, and yield parameters**

**Table 1. Analysis of variance of the effect of sulfhydryl thiourea applications on phenological and physiological parameters in wheat varieties under drought stress**

| **SOV** | **DF** | **PH** | **DW** | **LAI** | **LAD** | **CGR** | **NAR** | **PN** | **E** | **GS** | **WP** | **RWC** | **CHLa** | **CHLb** |
| --- | --- | --- | --- | --- | --- | --- | --- | --- | --- | --- | --- | --- | --- | --- |
| V | 3 | 0.0000 | 0.0000 | 0.0000 | 0.0000 | 0.0000 | 0.0000 | 0.0000 | 0.0000 | 0.0000 | 0.0000 | 0.0000 | 0.0000 | 0.0000 |
| S | 1 | 0.0000 | 0.0000 | 0.0000 | 0.0000 | 0.0000 | 0.0000 | 0.0000 | 0.0000 | 0.0000 | 0.0000 | 0.0000 | 0.0000 | 0.0000 |
| D | 1 | 0.0000 | 0.0000 | 0.0000 | 0.0000 | 0.0000 | 0.0000 | 0.0000 | 0.0000 | 0.0000 | 0.0000 | 0.0000 | 0.0000 | 0.0000 |
| V*S | 3 | 0.0101 | 0.3086 | 0.3514 | 0.0000 | 0.1624 | 0.3039 | 0.0000 | 0.0000 | 0.0004 | 0.0000 | 0.3929 | 0.2036 | 0.5236 |
| V*D | 3 | 0.8639 | 0.7696 | 0.3034 | 0.1288 | 0.0012 | 0.0065 | 0.0000 | 0.0002 | 0.0000 | 0.0000 | 0.8127 | 0.0704 | 0.6991 |
| S*D | 1 | 0.0000 | 0.0000 | 0.0000 | 0.0000 | 0.0000 | 0.0167 | 0.0000 | 0.0000 | 0.0000 | 0.0000 | 0.0000 | 0.0003 | 0.1397 |
| V*S*D | 3 | 0.0239 | 0.6639 | 0.8426 | 0.0085 | 0.6815 | 0.0056 | 0.0000 | 0.0000 | 0.0002 | 0.0011 | 0.2840 | 0.5781 | 0.3328 |

SOV=Source of variance, DF=Degree of freedom, V=Varieties, S= Sulfhydryl thiourea, D=Drought stress, PH=Plant height, DW=Dry weight, LAI=Leaf area index, LAD=Leaf area duration, CGR= Crop growth rate, NAR=Net assimilation rate, Pn=Photosynthetic rate, Tr=Transpiration rate, gs=Stomatal conductance, WP=Water potential, RWC=Relative water content.

**Table 2. Analysis of variance of the effect of sulfhydryl thiourea applications on biochemical and yield parameters in wheat varieties under drought stress**

| **SOV** | **DF** | **Pr** | **GB** | **Tph** | **SOD** | **POD** | **CAT** | **MDA** | **EL** | **NPT** | **NSS** | **NGS** | **TGW** | **BY** | **SY** | **HI** |
| --- | --- | --- | --- | --- | --- | --- | --- | --- | --- | --- | --- | --- | --- | --- | --- | --- |
| V | 3 | 0.0000 | 0.0000 | 0.0000 | 0.0000 | 0.0000 | 0.0000 | 0.0000 | 0.0000 | 0.0000 | 0.0000 | 0.0000 | 0.0000 | 0.0000 | 0.0000 | 0.0000 |
| S | 1 | 0.0000 | 0.0000 | 0.0000 | 0.0000 | 0.0000 | 0.0000 | 0.0000 | 0.0000 | 0.0000 | 0.0000 | 0.0000 | 0.0000 | 0.0000 | 0.0000 | 0.0000 |
| D | 1 | 0.0000 | 0.0000 | 0.0000 | 0.0000 | 0.0000 | 0.0000 | 0.0000 | 0.0000 | 0.0000 | 0.0000 | 0.0000 | 0.0000 | 0.0000 | 0.0000 | 0.0011 |
| V*S | 3 | 0.6632 | 0.5780 | 0.5986 | 0.0015 | 0.0001 | 0.1099 | 0.9635 | 0.9784 | 0.6482 | 0.9610 | 0.6862 | 0.8588 | 0.7109 | 0.1809 | 0.7074 |
| V*D | 3 | 0.0000 | 0.1980 | 0.0000 | 0.1980 | 0.3534 | 0.9559 | 0.0000 | 0.0000 | 0.5693 | 0.8430 | 0.1247 | 0.7293 | 0.6539 | 0.9466 | 0.8275 |
| S*D | 1 | 0.4852 | 0.0000 | 0.0196 | 0.0000 | 0.0000 | 1.0000 | 0.0000 | 0.0000 | 0.1365 | 0.0293 | 0.6172 | 0.0142 | 0.2125 | 0.1270 | 0.2790 |
| V*S*D | 3 | 0.0232 | 0.8901 | 0.8296 | 0.8901 | 0.3006 | 1.0000 | 0.7559 | 0.9424 | 0.9860 | 0.9199 | 0.4856 | 0.9273 | 0.9298 | 0.8682 | 0.8362 |

SOV=Source of variance, DF=Degree of freedom, Pr=Proline, GB=Glycine betaine, Tph=Total phenolics, SOD=Superoxide dismutase, POD=Peroxidase, CAT=Catalase, MDA=Malondialdehyde, EL=Electrolyte leakage, NPT=Number of productive tillers, NS=Number of seeds per spike, NGS=Number of grains per spike, TGW=Thousand grain weight, BY=Biological yield, SY=Seed yield, HI=Harvest index
